# Is hemorrhoidal surgery really unsafe in inflammatory bowel disease? Results from the HEAD-IBD multicenter study on behalf of IG-IBD

**DOI:** 10.1007/s10151-026-03394-6

**Published:** 2026-07-26

**Authors:** C. Felice, D. Pugliese, G. Gallo, G. L. Di Tanna, M. Ascolani, G. S. Sica, V. Bellato, M. Franco, F. Scaldaferri, L. Parisio, F. Colombo, A. Bondurri, C. Guerci, A. Aratari, S. Festa, F. Ascari, E. V. Savarino, U. Grossi

**Affiliations:** 1 Department of Medicine (DIMED), University of Padova, Padua, Italy; 2 Medicine 1, Ca’ Foncello University Hospital, Treviso, Italy; 3 Department of Translational Medicine and Surgery, Hospital Isola Tiberina Gemelli Isola, Catholic University of Rome, Rome, Italy; 4 Colorectal Surgery Unit, Faculty of Medicine and Surgery, IRCCS San Raffaele Scientific Institute, Vita-Salute University, Milan, Italy; 5 University of Applied Sciences and Arts of Southern Switzerland, Manno, Switzerland; 6 Gastroenterology Unit, Ca’ Foncello University Hospital, Treviso, Italy; 7 Department of Surgical Sciences, University of Rome Tor Vergata, Rome, Italy; 8 Medicina Interna e Gastroenterologia, UOS Malattie Infiammatorie Croniche Intestinali, IBD Unit, CEMAD - Fondazione Policlinico A. Gemelli IRCCS, Rome, Italy; 9 Dipartimento di Medicina e Chirurgia Traslazionale, Università Cattolica del Sacro Cuore, Rome, Italy; 10 L. Sacco University Hospital General Surgery II, Milan, Italy; 11 Department of Gastroenterology, IBD Unit, San Filippo Neri Hospital, ASL ROMA 1, Rome, Italy; 12 UOC Chirurgia Generale, AUSL Modena, Modena, Italy; 13 Department of Surgery, Oncology and Gastroenterology, University of Padova, Padua, Italy; 14 Proctology and Pelviperineology Unit, Humanitas Gavazzeni and Castelli, Bergamo, Italy

**Keywords:** Hemorrhoidal disease, Inflammatory bowel disease, Crohn’s disease, Ulcerative colitis, Hemorrhoidectomy, Hemorrhoidopexy

## Abstract

**Background:**

Surgical management of hemorrhoidal disease (HD) in patients with IBD remains controversial because of concerns about impaired outcomes. This study aimed to evaluate the safety and IBD-related outcomes of surgical treatments for HD in patients with IBD and to explore factors potentially associated with postoperative complications and IBD flare.

**Methods:**

HEAD-IBD is a retrospective cohort study promoted by the Italian Group for the study of IBD (IG-IBD). Adults with established ulcerative colitis (UC) or Crohn’s disease (CD) who underwent surgery for HD between 2004 and 2024 and had at least 12 months of follow-up were included. Surgical techniques, 1-, 6-, and 12-month complications, IBD flares (6 months), new onset perianal disease, and continence outcomes were analyzed.

**Results:**

A total of 38 patients were included (21 UC, 17 CD), 55% male. Clinically active disease at the last gastroenterology visit prior to surgery was present in 24% of UC and 41% of patients with CD. Open excisional hemorrhoidectomy was the most frequently performed procedure in both groups (66% UC, 73% CD), while non-excisional techniques were used in approximately one-third of cases. No 30-day or 6-month surgical complications occurred in patients with CD. Postoperative surgical complications were observed exclusively in patients with UC (5/21, 24%) and were mostly mild. Within 6 months after surgery, IBD flares occurred in 6 patients (16%), proportionally comparable between CD and UC. During overall follow-up, new-onset perianal disease developed in 5 patients (13%), predominantly in CD (4/5 cases), while new-onset incontinence was observed in 2 (5%) patients, both managed conservatively.

**Conclusions:**

In this largest European multicenter cohort, surgical treatment of HD in IBD was associated with low morbidity and preservation of anorectal function. These findings support a more individualized approach and underscore the need for prospective studies.

## Introduction

Hemorrhoidal disease (HD) poses a substantial clinical burden worldwide, with a pooled point prevalence of approximately 26%, varying according to population characteristics and diagnostic approach [1]. In patients with inflammatory bowel disease (IBD), HD represents a frequent and clinically relevant anorectal condition, and recent data suggest a modest causal association between Crohn’s disease (CD) and HD, while evidence for ulcerative colitis (UC) appears less consistent [2].

To date, there is no consensus in the scientific literature regarding the exact indications for surgery for HD in patients with IBD [3]. While some authors recommend that surgery should only be considered in the absence of active disease, others refrain from providing specific guidance. Moreover, it remains uncertain whether more recently developed techniques may offer advantages over excisional hemorrhoidectomy. The current literature is limited to relatively small case series, with the largest series published in 2018 including 70 American patients with IBD who underwent hemorrhoidal surgery [4]. A subsequent systematic review analyzed 10 retrospective studies including 222 patients [5]. Most studies lacked information on the interval between surgery and the onset of complications. Operative treatments included open or closed hemorrhoidectomy (70%), rubber band ligation (18%), excision or incision of thrombosed hemorrhoid (6%), and Doppler-guided hemorrhoidal artery ligation (DG-HAL, 6%). The pooled prevalence of complications was 9% (95% CI 3–16%), with a more than two-fold higher rate in patients with CD compared to UC (11 vs. 5%, respectively). The authors concluded that further studies should determine whether advantages in terms of safety and postoperative recovery with the use of non-excisional techniques (e.g., DG-HAL) can be obtained in this patient population.

Given the frequency of hemorrhoidal surgery in routine practice, variation in operative approach across centers persists, particularly for patients with IBD.

The primary objective of this study was to determine the safety and IBD-related outcomes of surgical treatments for HD in a national multicenter cohort of patients with IBD. The secondary aim was to identify factors that may affect clinical and surgical outcomes.

## Methods

This is a 20-year, retrospective, multicenter cohort study assessing the safety and IBD-related outcomes of surgical treatments for HD in patients with IBD. All consecutive eligible surgical patients at each center during the study period were screened for inclusion.

### Setting

All patients with an established diagnosis of IBD who underwent surgery for HD from December 2004 to March 2024 and followed up for at least 1 year postoperatively were retrospectively included. Data on patient demographics and clinical characteristics, operative details, and clinical outcomes were collected.

The surgical outcomes included length of hospital stay, 30-day readmission, and postoperative complications at short- (30 days), medium- (6 months) and long-term (12 months) follow-up.

The clinical outcomes included IBD flare, defined according to standard scores (i.e., partial Mayo score for UC [6] and Harvey–Bradshaw Index (HBI) for CD [7]) and need for therapeutic change or dose escalation during the 6 months after surgery, new onset of perianal disease, anal continence.

Data were entered onto a secure anonymous online database. The main results publication was prepared in accordance with guidelines set by the STROBE (Strengthening the Reporting of Observational Studies in Epidemiology) statement for observational studies [8].

### Participants

Inclusion criteria were age of 18 years and above, with an established diagnosis of CD or UC at the time of surgery for HD, including hemorrhoidectomy (e.g., open, closed, or submucosal), transanal hemorrhoidal dearterialization, mucopexy, hemorrhoidal laser procedure, stapled hemorrhoidopexy, who completed at least 1-year postoperative follow-up.

Patients who underwent office-based treatments (e.g., sclerotherapy injection, rubber band ligation), or sole skin tag removal, those diagnosed with IBD after receiving surgery for HD or with IBD unclassified (IBD-U), and those with < 1 year of postoperative follow-up were excluded.

### Data collection

Clinical charts were reviewed in a retrospective fashion to obtain relevant factors including type of center (academic, non-academic teaching, non-teaching); volume of surgery for HD in the study period; number of surgeons involved; number of operations for HD per surgeon already performed; diagnosis of either CD or UC; duration of IBD; medications for IBD at the time of surgery; other comorbidities or medications, the Montreal classification of the patient’s IBD [9], IBD clinical activity at the last clinic visit before surgery, according to the partial Mayo score (remission ≤ 2 with no subscore > 1) and the HBI (remission ≤ 4) for UC and CD, respectively, time from the last IBD clinic visit to surgery. Because of the retrospective design, disease activity was classified according to the most recent gastroenterology assessment preceding surgery, which was considered the baseline disease activity for all analyses. Steroids and immunomodulators were considered positive if the last dose was taken within the 4 weeks before surgery, while biologics were considered positive if the last dose was received within the 12 weeks before surgery. If the patient had endoscopy performed within 1 year of the intervention, the report was reviewed to document endoscopic mucosal disease activity. Perianal disease was defined as the presence of any of the following at the time of intervention: skin tags, perianal abscess, anal fissure, anal stricture or fistula. Collected data also included details on the symptoms and Goligher grade of HD, the specific surgical intervention performed, 30-day readmission ± the need for repeat intervention, medium- (6 months) and long-term (12 months) surgical morbidity, including the development of an anal canal stricture or the requirement of proctectomy, and the date of last follow-up. IBD flare was defined by a partial Mayo score higher than 2 for patients with UC and a HBI higher than 4 for patients with CD who were in clinical remission at baseline, and/or need for therapeutic changes (dose optimization or drug change). For patients without clinical remission at baseline, a worsening of symptoms requiring a therapeutic change was also considered as a flare; anal continence status was determined using the St. Marks incontinence score at last follow-up [10].

Clinical data were collected anonymously by the investigators in a specific eCRF database inside the IG-IBD registry, created specifically for this study.

The study was approved by the ethics committee of the coordinating center (n. 1252/CE Marca). Approval was subsequently obtained from the local ethics committees of all participating centers. The study was conducted in accordance with the principles of the Declaration of Helsinki and national regulatory requirements.

### Statistical analysis

Values are expressed as median and interquartile range. The Mann–Whitney *U* test was used for continuous variables, and the chi-square test or Fisher’s exact test was used for categorical variables. To explore factors potentially associated with postoperative surgical complications, a binary logistic regression model was performed with the occurrence of postoperative complications as the dependent variable and age at surgery, time interval between the last IBD clinical assessment and surgery, and disease activity score (partial Mayo score for UC) as independent covariates. Since all postoperative complications occurred exclusively in patients with UC, this analysis was performed post hoc in the UC subgroup only.

A separate multivariable binary logistic regression model was conducted to investigate factors associated with postoperative IBD flare within 6 months. Gender, disease duration, clinically active IBD at the last gastroenterology visit prior to surgery, and use of advanced therapy were included as independent variables.

Given the limited number of events, both regression analyses were considered exploratory. Statistical significance was set at a *p* value of < 0.05. All statistical analyses were performed using Stata version 17 and R.

## Results

### Participants

A total of 40 patients with IBD who underwent surgery for HD were initially identified. Two patients with IBD-U were excluded from stratified analyses, leaving 38 patients for the final analysis, of whom 21 (55%) had UC and 17 (45%) CD. Overall, 55% were male.

Baseline characteristics are summarized in Table 1. Patients with UC were significantly older at the time of surgery compared with those with CD (median age 49 [IQR 13] vs. 37.5 [IQR 24] years, *p* = 0.016). Disease duration was comparable between groups. A significantly higher proportion of patients with CD were active smokers (*p* = 0.001), and previous abdominal IBD surgery was more frequent in CD than UC (35% vs. 5%, *p* = 0.031).

**Table 1:** Patients’ characteristics

	Crohn’s disease (CD) (*n* = 17)	Ulcerative colitis (UC) (*n* = 21)	*p*
Female, *n* (%)	8 (47)	9 (43)	0.999
Age (years), median (IQR)	37.5 (24)	49 (13)	0.016*
Disease duration (years), median (IQR)	5.5 (17)	8 (21)	0.562
Smoking status			0.001*
Active smokers	10 (59)	1 (5)	
Former smokers	2 (12)	11 (52)	
Non-smokers	5 (29)	9 (43)	
Ulcerative colitis localization, *n* (%)	–		–
E1 ulcerative proctitis		3 (14)	
E2 left-sided colitis		11 (52)	
E3 pancolitis		7 (33)	
Crohn’s disease localization, *n* (%)		–	–
L1 ileal	6 (35)		
L2 colonic	5 (29)		
L3 ileocolonic	6 (35)		
L4 upper disease	1 (6)		
Crohn’s disease behaviors, *n* (%)		–	–
B1 non-stricturing, non-penetrating	9 (53)		
B2 stricturing	7 (41)		
B3 penetrating	1 (6)		
History of perianal Crohn’s disease, *n* (%)	4 (24)	–	–
Previous abdominal IBD surgery, *n* (%)	6 (35)	1 (5)	0.031*
Comorbidities, *n* (%)	6 (35)	8 (38)	0.859
Therapy at the time of surgery, *n* (%)			
Topical therapy	0 (0)	1 (5)	0.999
Oral mesalamine/sulfasalazine	3 (18)	0 (0)	0.081
Steroids	3 (18)	1 (5)	0.307
Immunosuppressors	1 (6)	1 (5)	0.999
Advanced therapy	8 (47)	2 (9.5)	0.023*
Anti-TNF	8	1	
Anti-integrin	0	0	
Anti-IL-12/23	0	1	
Time interval between IBD evaluation and surgery (months), median (IQR)			
Clinical	3.5 (2.9)	2.5 (5.7)	0.371
Endoscopic	5.0 (2.5)	6.3 (9.3)	0.857
Latest partial Mayo score (endoscopic) before surgery, *n* (%)	–		–
0–1		13	
2–3		7	
Clinically active IBD at last GI visit before surgery, *n* (%)	7 (41)	5 (24)	0.543
Goligher grade, *n* (%)			0.208
I	3 (18)	1 (5)	
II	2 (12)	4 (19)	
III	9 (53)	7 (33)	
IV	3 (18)	9 (43)	
Perianal disease at the time of surgery, *n* (%)	3	2	0.640
Fistula	0	1	
Fistula and stricture	1	0	
Fissure	0	1	
Abscess and fistula	2	0	
Surgical procedures, *n* (%)			0.411
Open hemorrhoidectomy	12 (71)	14 (67)	
Closed hemorrhoidectomy	0	1 (5)	
Dearterialization and/or mucopexy	5 (29)	4 (19)	
Stapled hemorrhoidopexy	0	2 (9)	
Postoperative length of stay (days), median (IQR)	1 (2)	1 (1)	0.517
Follow-up (months), median (IQR)	46 (99)	54 (85)	0.691

*Statistically significant (*p* < 0.05)

Regarding IBD activity at the last gastroenterology visit prior to surgery, clinically active disease was present in 24% of patients with UC and 41% of patients with CD. Among patients with UC, partial Mayo scores and endoscopic activity were variably distributed, while patients with CD showed a median HBI of 5 (IQR 3–8). The median time interval between the last clinical and endoscopic IBD evaluation and surgery did not differ significantly between patients with UC and patients with CD.

HD severity differed between cohorts: grade IV HD was more frequent in patients with UC than in patients with CD (43% vs. 18%), although this difference did not reach statistical significance. Perianal disease at the time of surgery was observed in patients with UC and patients with CD, with no significant differences between groups.

### Surgical procedures

Surgical details are reported in Table 2. Open hemorrhoidectomy was the most frequently performed procedure in both groups (66% in UC and 73% in CD). Doppler-guided hemorrhoidal artery ligation (DG-HAL) and/or mucopexy was performed in approximately one-third of patients in each group. Stapled hemorrhoidopexy was performed exclusively in patients with UC (10%). Median postoperative length of stay was 1 day in both cohorts.

**Table 2:** Postoperative complications and clinical outcomes

Patient ID^a^	Age (years)	Gender	IBD	Montreal	Active IBD prior to surgery	Goligher grade	Surgery	Complication	Time to complication	Treatment
Surgical outcomes										
110–3	42	Female	UC	E2	No	IV	OEH	Soiling	1 day	Conservative
111–6	53	Male	UC	E3	No	IV	CEH	Abscess	3 days	Antibiotic therapy
117–1	45	Female	UC	E2	Yes	IV	OEH	Fistula and abscess	12 days	Drainage and seton
117–2	49	Male	UC	E2	Yes	III	SH	Soiling	1 day	Conservative
117–4	39	Male	UC	E2	Yes	III	OEH	Soiling and rectal bleeding	1 day	Conservative
Clinical outcomes										
Flare within 6 months from surgery										
104–3	49	Male	UC	E2	Yes	IV	SH	Flare	7.9 weeks	Topical steroid + mesalamine dose optimization
105–1	44	Female	UC	E3	Yes	III	OEH	Flare	4.3 weeks	Oral steroid
113–4	37	Male	CD	L1 + L4/B3	No	III	SH	Flare	18.3 weeks	Infliximab optimization
117–1	45	Female	UC	E2	Yes	IV	OEH	Flare	1.7 weeks	Oral steroid + mesalamine dose optimization
311–1	42	Female	CD	L1/B2	Yes	III	OEH	Flare	11.1 weeks	Steroid dose optimization
311–4	22	Female	CD	L3/B1	Yes	IV	OEH	Flare	20.6 weeks	Ustekinumab
New onset perianal disease										
104–4	57	Female	CD	L1/B2	No	I	OEH	Abscess	12.6 years	Drainage
117–1	45	Female	UC	E2	Yes	IV	OEH	Fistula and abscess	9 months	Drainage and fistulotomy
117–3	19	Male	CD	L3/B1p	No	III	OEH	Fistula and fissure	3.5 years	Fistulotomy
311–1	42	Female	CD	L1/B2	Yes	III	OEH	Inflamed skin tags	13 months	Topical ointment
311–4	22	Female	CD	L3/B1	Yes	IV	OEH	Fistula and abscess	16 months	Antibiotic therapy
New onset incontinence										
113–4	37	Male	CD	L1 + L4/B3	No	III	SH	Incontinence	4.3 months	Conservative
117–1	45	Female	UC	E2	Yes	IV	OEH	Incontinence	5.9 years	Conservative

*CD* Crohn’s disease, *UC* ulcerative colitis, *OEH* open excisional hemorrhoidectomy, *SH* stapled hemorrhoidopexy, *CEH* closed excisional hemorrhoidectomy

^a^Patients may appear more than once if multiple events occurred

**Fig. 1:**
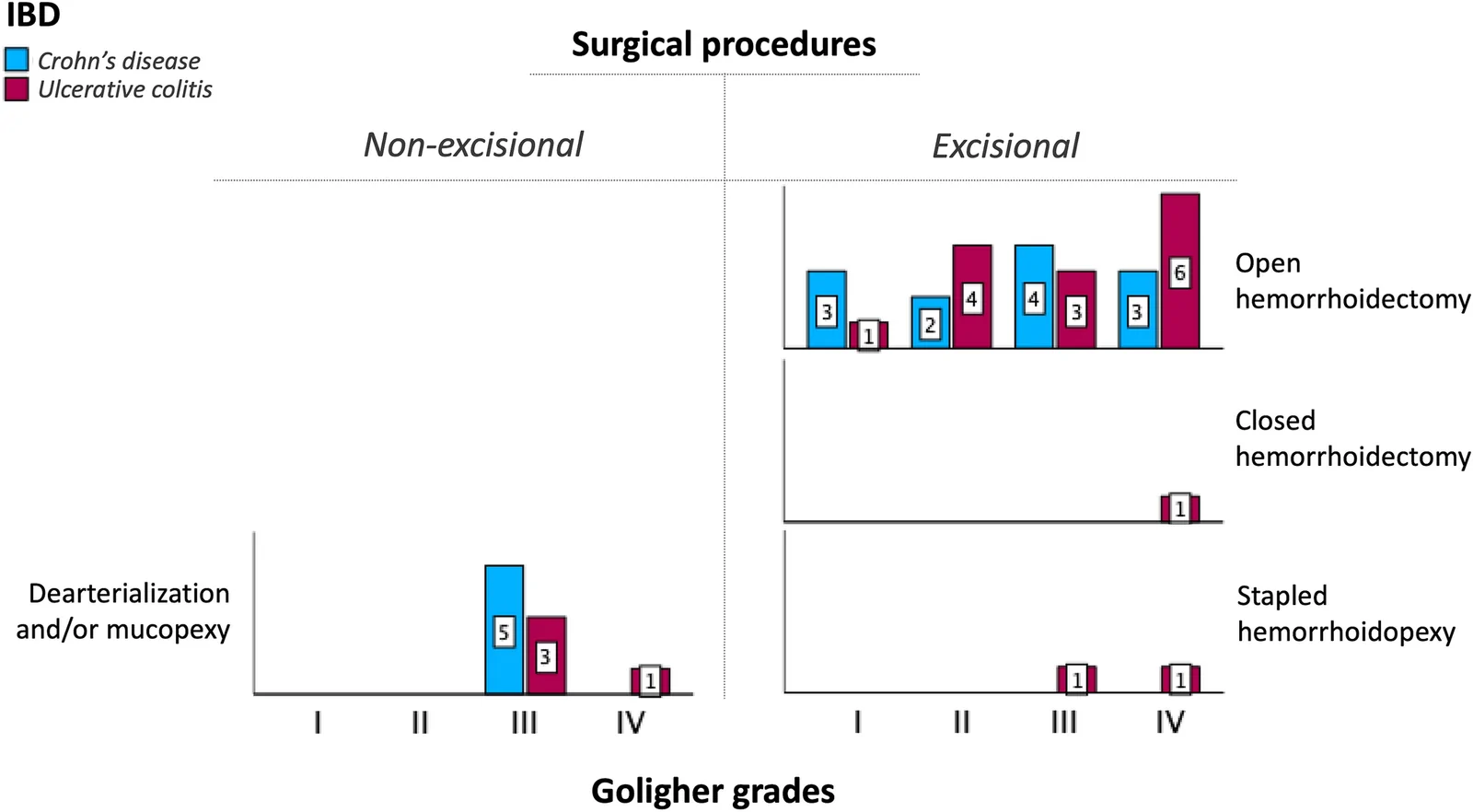
Distribution of surgical procedures for hemorrhoidal disease according to Goligher grade and inflammatory bowel disease subtype. In selected patients with Goligher grade I–II disease, a limited open excisional hemorrhoidectomy involving fewer than three piles was performed

Figure 1 depicts the distribution of surgical procedures by Goligher grade and IBD subtype, grouped as excisional (open/closed hemorrhoidectomy and stapled hemorrhoidopexy) and non-excisional (DG-HAL and/or mucopexy). Excisional surgery predominated across grades in both cohorts, whereas non-excisional techniques were mainly performed in patients with grade III disease.

### Surgical outcomes

Surgical complications are detailed in Table 2. No 30-day or 6-month surgical complications were observed among patients with CD. In contrast, postoperative surgical complications occurred exclusively in patients with UC (5/21, 24%). These included perianal abscess (*n* = 1), fistula and abscess (*n* = 1), and early postoperative soiling with or without rectal bleeding (*n* = 3). All events occurred within 12 days after surgery. Most surgical complications occurred in patients with advanced HD, with 4 out of 5 cases (80%) having Goligher grade IV HD. At 12-month follow-up, no cases of anal canal stricture or need for proctectomy were recorded.

In exploratory logistic regression analysis restricted to patients with UC, age at surgery, interval between last IBD clinical assessment and surgery, and baseline partial Mayo score were not significantly associated with the occurrence of postoperative surgical complications.

### Clinical outcomes

Within 6 months after surgery, postoperative IBD flares occurred in 6 patients (16% of the cohort), including 3 patients (18%) with CD and 3 (14%) with UC (*p* = 0.778). Flares were managed with treatment optimization, including corticosteroids, mesalamine dose adjustment, biologic escalation, or initiation of advanced therapy (Table 2).

In exploratory multivariable logistic regression analysis, none of the investigated covariates (gender, disease duration, clinically active IBD at the last gastroenterology assessment before surgery, and use of advanced therapy) were independently associated with postoperative flare within 6 months.

New-onset perianal disease during overall follow-up was observed in 5 patients (13%), including 4 with CD and 1 with UC. These events comprised abscess (*n* = 1), fistula with or without abscess or fissure (*n* = 3), and inflamed skin tags (*n* = 1). The median time to onset was 16 months after surgery (range 9 months–12.6 years). Management included drainage, fistulotomy, antibiotic therapy, or conservative treatment.

Regarding anal continence, two patients (5%) developed new-onset incontinence or persistent soiling during long-term follow-up, both managed conservatively. No patient required major surgical intervention for continence impairment.

## Discussion

The management of HD in patients with IBD remains controversial, largely due to historical concerns regarding postoperative complications, impaired wound healing, and the potential risk of triggering disease flares. These concerns largely originate from small historical case series conducted before the widespread availability of optimized immunomodulatory and biologic therapy, reporting severe wound-healing complications and subsequent proctectomy in patients with CD [5]. Operative reluctance has been perpetuated over time by the absence of high-quality prospective data, ultimately shaping contemporary guidelines that still recommend extreme caution when considering excisional hemorrhoidectomy in CD [11].

A subsequent systematic review with proportional meta-analysis confirmed a higher complication rate in CD compared with UC, but also underscored the overall poor quality and heterogeneity of the available evidence [5]. More recently, a narrative review questioned the validity of this “old dogma,” highlighting the limited strength of the evidence supporting such recommendations [12].

In this national multicenter cohort, surgical treatment for HD in patients with IBD was associated with low postoperative morbidity and preservation of anorectal function at 12-month follow-up. Notably, no early or mid-term postoperative complications were observed in patients with CD, whereas in patients with UC few early or mid-term complications occurred but were mostly mild, including perianal abscess and early transient soiling with or without rectal bleeding. Importantly, postoperative IBD flares within 6 months occurred in a minority of patients (16%), proportionally comparable between CD and UC, and were managed with medical optimization. At long-term follow-up, new-onset incontinence was observed in two patients (5%), one with CD and one with UC, both managed conservatively. Notably, no cases of anal stricture, need for proctectomy, or permanent functional impairment were recorded in either group. In a dedicated UC cohort, Lightner et al. [13] reported only two early surgical complications requiring reoperation for bleeding (4.9%), with no cases of nonhealing, stricture, diversion, proctectomy, or pouch dysfunction; however, IBD flares and functional outcomes were not systematically assessed.

Taken together, these findings support a reappraisal of the traditional paradigm, suggesting that surgical treatment of HD can be performed safely in both UC and CD, even in the presence of clinically active disease in a subset of patients. Importantly, poorer outcomes have been historically linked to procedures performed before CD was recognized, likely reflecting uncontrolled inflammation and lack of optimized medical therapy—supporting the rationale for excluding patients diagnosed with IBD only after hemorrhoidal surgery [5, 11, 12]. It should also be acknowledged that the favorable outcomes observed in our cohort may, at least in part, reflect careful patient selection.

A relevant aspect of our study is the systematic assessment of IBD activity prior to surgery using standardized clinical indices (partial Mayo score and HBI), a feature that has been inconsistently reported in previous series (Table 3). In our cohort, clinically active disease was present in nearly one-quarter of patients with UC and in 41% of patients with CD at the last gastroenterology visit prior to surgery, yet this did not translate into an increased rate of surgical complications or severe disease exacerbation.

**Table 3:** Previous studies on hemorrhoidal disease in inflammatory bowel disease (IBD)

First author	Year	Country	Study period (years)	*N*^a^	CD	UC	Active IBD prior to surgery	Type of operation	Postoperative complications (*N*)		Average follow-up (months)
									CD	UC	
Jeffery [17]	1977	UK	40	20	4	16	NR	OEH	1	3	480
Hughes [18]	1978	UK	6	2	2	0	NR	OEH	1	–	72
Keighley [19]	1986	UK	1	2	2	0	NR	RBL	1	–	12
Wolkomir [20]	1993	USA	15	17	17	0	0	CEH^b^	4	–	137
Karin [21]	2012	Israel	4	13	13	0	NR	DGHAL	3	–	18
D’Ugo [22]	2013	Italy	8	9	9	0	0	Mixed^d^	1	–	37
Koh [23]	2015	USA	14	9	9	0	1	OEH	0	–	28
Lee [24]	2017	S. Korea	11	44	0	44	NR	OEH	–	0	NR
McKenna [4]	2019	USA^c^	17	70	27	43	NR	Mixed^e^	2	3	29
Lightner [11]	2020	USA^c^	24	36	36	0	NR	OEH	4	–	31.5
Lightner [13]	2020	USA^c^	24	41	0	41	NR	OEH	–	2	48
Alam [15]	2021	France	7	25	13	12	0	Mixed^f^	4	2	15
Zakaria [16]	2024	Egypt	1	26	12	14	0	Mixed^g^	4	3	6
Present study	2026	Italy^c^	20	38	17	21	17	Mixed	0	5	54

*CD* Crohn’s disease, *UC* ulcerative colitis, *OEH* open excisional hemorrhoidectomy, *RBL* rubber band ligation, *CEH* closed excisional hemorrhoidectomy, *DGHAL* Doppler-guided hemorrhoidal artery ligation, *NR* not reported

^a^Total number of patients already diagnosed with IBD at the time of surgery for HD, which may not correspond to the total number of study patients

^b^Less than 3-quadrant in 5/17 (29%) patients

^c^Multicenter

^d^Included OEH (*n* = 6), CEH (*n* = 1), RBL (*n* = 2)

^e^Included RBL (*n* = 35), OEH or CEH (*n* = 21), excision or incision of thrombosed hemorrhoid (*n* = 14)

^f^Included mono-hemorrhoidectomy (*n* = 3), tripedicular hemorrhoidectomy (*n* = 16), artery ligations with mucopexy (*n* = 6), artery ligations with mucopexy followed by tripedicular hemorrhoidectomy for recurrence (*n* = 1)

^g^Included laser hemorrhoidoplasty (*n* = 13) and OEH (*n* = 13)

This finding challenges the traditional notion that hemorrhoidal surgery should be strictly avoided outside complete disease remission and suggests that mild-to-moderate activity, when adequately characterized and monitored, may not represent an absolute contraindication. However, given the retrospective design and limited sample size, this observation should be interpreted cautiously and warrants confirmation in larger prospective studies.

Open excisional hemorrhoidectomy remained the most performed procedure in both UC and CD, consistent with current recommendations that reserve this approach for advanced prolapse, thrombosis, or mixed disease refractory to less invasive treatments [14]. Non-excisional techniques, particularly DG-HAL and/or mucopexy, were used in approximately one-third of patients in both groups, while stapled hemorrhoidopexy was selectively performed in patients with UC only.

As shown in Fig. 1, excisional procedures predominated in both UC and CD. Notably, a subset of patients with Goligher grade I–II disease underwent targeted open excisional hemorrhoidectomy, typically limited to a single symptomatic pile, reflecting symptom-driven decision-making (most commonly recurrent bleeding) rather than prolapse severity alone. Similar selective excisional approaches, including mono-hemorrhoidectomy, have been previously reported in carefully selected patients with IBD in remission [15], reinforcing the concept that surgical strategy may deviate from a strictly grade-based algorithm when symptoms are disabling and conservative measures fail.

Although the study was not powered to compare techniques, the overall low complication rate across different surgical approaches suggests that procedure selection tailored to disease severity, hemorrhoidal grade, and local expertise may be more relevant than the specific technique itself. Importantly, no signal of increased morbidity was observed with excisional procedures, historically considered higher risk in IBD populations.

When contextualized within the existing literature, the evidence supporting hemorrhoidal surgery in IBD remains largely based on retrospective case series, with substantial heterogeneity in patient selection, operative techniques, and outcome reporting [5]. Notably, the randomized trial by Zakaria et al. [16] represents an important attempt to compare patient-reported recovery and short-term surgical outcomes between laser hemorrhoidoplasty (a non-excisional approach) and conventional excisional hemorrhoidectomy, but its interpretability for IBD-specific decision-making is limited by the single-center design, small subgroup sizes (particularly for CD and UC), inclusion of “nonspecific colitis,” short follow-up, and the lack of granular characterization of IBD phenotype and activity (e.g., Montreal classification, validated activity indices such as partial Mayo or HBI, and objective endoscopic assessment). Moreover, the trial primarily focuses on short-term surgical outcomes without standardized reporting of postoperative IBD flares or treatment escalation.

Our findings are consistent with more recent series, including those by Lightner et al. [13], which demonstrated acceptable safety profiles for excisional hemorrhoidectomy in patients with UC, even in complex surgical settings.

Taken together, these data suggest that hemorrhoidal surgery should not be considered universally contraindicated in patients with IBD. Instead, a multidisciplinary approach involving gastroenterologists and colorectal surgeons, careful preoperative assessment of disease activity, and appropriate patient selection appear crucial to achieving favorable outcomes. The presence of IBD alone should not preclude surgical intervention for symptomatic HD when conservative measures fail.

Despite the relatively small sample size, this study has several important strengths. First, it provides a detailed phenotypic characterization of IBD, including Montreal classification and systematic assessment of clinical activity using validated indices (partial Mayo score and HBI), features that have been inconsistently reported in previous series. Second, outcomes were predefined and distinctly categorized as surgical and clinical, allowing separate evaluation of early postoperative morbidity and disease-related events. Importantly, postoperative IBD flares within 6 months were captured according to protocol, and long-term outcomes included new-onset perianal disease and continence status, domains that have rarely been systematically assessed in prior studies.

### Limitations

This study has several limitations. Its retrospective design introduces the possibility of selection and information bias, and the relatively small sample size limits the ability to detect rare adverse events or perform robust subgroup analyses by surgical technique. Endoscopic disease activity was not uniformly available for all patients, and histological activity was not systematically assessed. Additionally, functional outcomes were evaluated using continence scores at follow-up, but baseline continence status was not available for all patients.

## Conclusions

In this multicenter cohort, surgical treatment of HD in selected patients with IBD was associated with low postoperative morbidity, minimal impact on disease course, and preservation of anorectal function. Although these findings are reassuring, they should be interpreted cautiously given the retrospective design, the limited sample size, and the small number of patients with clinically active disease. Therefore, our results support an individualized, multidisciplinary approach to the surgical management of HD in patients with IBD, rather than broad conclusions regarding its safety. Larger prospective studies are needed to better define optimal timing, patient selection, and procedure choice. In this context, the ongoing HEAD-IBD II study (ClinicalTrials.gov identifier NCT06638814) has been designed as a multicenter European prospective cohort to systematically evaluate the safety and patient-centered outcomes of surgical treatment for HD in IBD over a 12-month follow-up.

## Data Availability

The data that support the findings of this study are available from the corresponding authors upon reasonable request.
